# Assessment of the Functional Properties of 316L Steel Alloy Subjected to Ion Implantation Used in Biotribological Systems

**DOI:** 10.3390/ma14195525

**Published:** 2021-09-24

**Authors:** Katarzyna Piotrowska, Monika Madej, Dariusz Ozimina

**Affiliations:** Department of Mechatronics and Mechanical Engineering, Kielce University of Technology, al. Tysiąclecia Państwa Polskiego 7, 25-314 Kielce, Poland; mmadej@tu.kielce.pl (M.M.); ozimina@tu.kielce.pl (D.O.)

**Keywords:** biomaterials, friction, hardness, ion implantation, wear

## Abstract

Clinical trials conducted in many centres worldwide indicate that, despite advances made in the use of biomaterials for medical applications, tribocorrosive wear remains a significant issue. The release of wear residue into body fluids can cause inflammation and, as a result, implant failure. Surface modification is one of the methods used to improve the mechanical, tribological, and fatigue properties of biomaterials. In this article, the authors investigated the impact of ion implantation on improving the functional properties of implant surfaces. This paper presents morphology, geometric surface structure, hardness, and tribological test results for layers obtained by ion implantation with nitrogen and oxygen ions on alloy 316L. The surface morphology and thickness of the implanted layer were examined using scanning microscopy. Atomic force microscopy was used to evaluate the geometric structure of the surface. Instrumented indentation was used to measure nanohardness. Model tribo tests were carried out for reciprocating motion under conditions of dry friction and lubricated friction with Ringer’s solution. The tribological tests showed that the implanted samples had a lower wear than the reference samples. Nitrogen ion implantation increased the hardness of 316L steel by about 45% and increased it by about 15% when oxygen ions were used.

## 1. Introduction

The increase in the number of osteoarticular injuries has intensified research on biotribology and biomaterials [1]. As a result, new materials and advanced surface treatments are being developed and tested with the aim of improving tribological properties, corrosion resistance, and biocompatibility.

Clinical trials conducted worldwide show that corrosive and mechanical wear on the implant surface remains a major issue despite ongoing efforts. The release of metal wear particles into body fluids can lead to metallosis in soft tissues and the formation of pseudotumors [2,3,4]. Inflammatory reactions that develop due to corrosion and metallosis caused by toxic and allergic reactions are due to a change in the body fluid pH resulting from the presence of metal wear debris. Implants remain in constant contact with body fluids, which have a high concentration of chloride ions that are strongly corrosive to metals. The process is intensified by the presence of proteins and amino acids in body fluids. Under normal conditions, body fluids have a pH in the range of 7.35–7.45. Implantation modifies neutral pH, which drops to about 5.2 and returns to normal after approximately 14 days [5,6].

Metals and alloys used in implantation should have a good corrosion resistance, biotolerance (non-toxicity), an appropriate chemical composition, a fine-particle structure, high strength, no tendency to form clots, and easy mechanical processing [7,8,9,10]. The most commonly used implant materials are alloyed steels characterized by excellent strength parameters, a high biocompatibility allowing implant–bone osseointegration, a structure that ensures high corrosion resistance, and very good technological properties that enable the use of advanced treatments. The property enhancement of implant steels, including AISI 316, aims to increase corrosion resistance under a body fluid environment. This is how 316L steel appeared on the market [11,12,13]. The corrosion resistance of 316L stainless steel was significantly increased by adding molybdenum. As a result, the chromium compounds stabilized and formed a passive layer on the steel surface in the presence of chloride ions.

To mitigate the adverse effects of wear debris on the human body, researchers typically alter the functional properties of implanted materials by modifying the implant surface layer [14,15]. This study investigated nitrogen and oxygen ion implantation, aiming at improving the functional properties of 316L steel for biomedical applications [16,17,18,19]. The importance of this method in surface engineering has been confirmed by a significant number of studies and publications [20,21]. Although initially used only in nuclear physics, over time the method has found application in various industries, including electronics, materials, and medicine [22,23,24,25,26]. 

**Figure 1:**
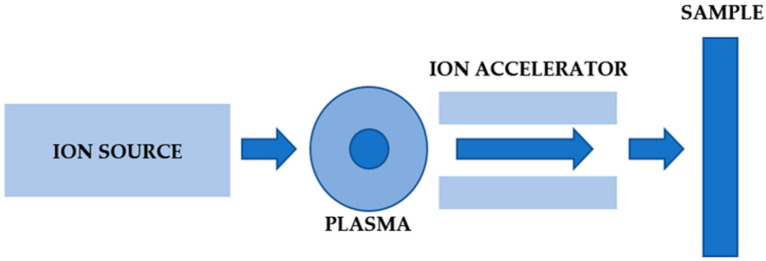
Schematic diagram of an advanced metal ion implantation method.

Ion implantation is a process in which atoms of any element are implanted into the core of the substrate material at a high kinetic energy. The atoms of the doped elements are ionized in the ion source, then accelerated in an electric field to energies ranging from several keV to several MeV. As a result of this process, the atoms of the base material “mix” with the implanted ions. The implanted ions penetrate the material core to a depth of 0.01 to 1 µm [25]. Figure 1 shows a schematic diagram of the implantation process.

An implantation profile is a measure of the implanted layer thickness. It is a curve defining the distribution of implanted ions at different depths in the base material. The thickness of the resulting layer depends on the properties (atomic weight) of the doped and doping materials, ion energy, ion current density, and doping duration. Due to the kinetic nature of the process, any material can be doped with virtually any element, which enables materials with the assumed functional properties to be obtained. As a result, it is possible to obtain a very high dopant concentration (up to 50%). In addition, the process is characterized by a very high purity and can be carried out at low temperatures [26].

## 2. Materials and Methods

Type 316L alloy with the chemical composition shown in Table 1 was chosen for examination. This steel type is characterized by a high corrosion resistance in an environment of weak organic acids. However, it is susceptible to pitting and crevice corrosion in the presence of chloride ions. Therefore, to improve its functional properties, ion implantation with nitrogen and oxygen ions was applied.

The grinding and polishing of sample surfaces were key steps in their preparation. Another important factor was the appropriate selection of the machining parameters, as these affect the performance of the components. The 20 × 20 × 5 mm plate-shaped samples were ground using a Pace Technologies grinder. Silicon carbide sandpaper with a grit size ranging from 120 to 2500 µm was used. The final step was polishing with cloths using a 1 µm grade diamond paste. After grinding and polishing, the surface roughness values were in the range of Ra = 1.5–2 μm. Prior to ion implantation, the samples were cleaned ultrasonically in ethyl alcohol and then implanted with nitrogen and oxygen ions using disruptive technology, hardion^TM^ by Idonus (Hauterive/Neuchâtel, Switzerland). The ion dose was 5 × 10^17^ N^+^/cm^2^ and 5 × 10^17^ O^+^/cm^2^, with an energy of 35 keV.

**Figure 2:**
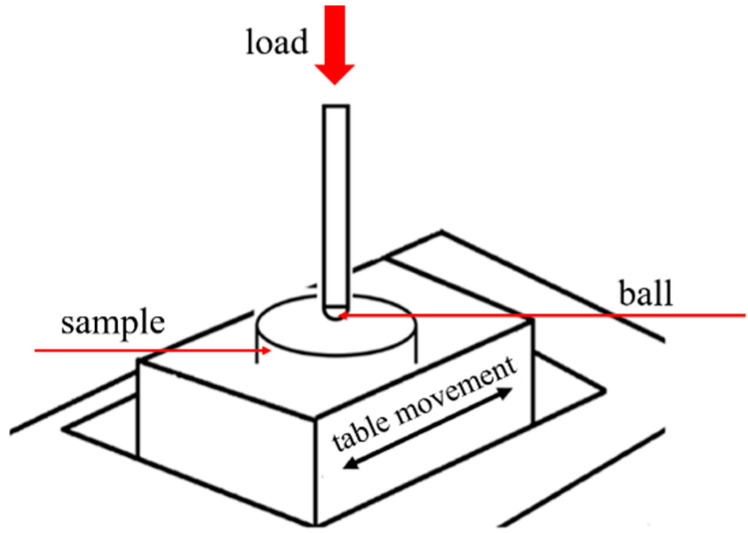
Ball-on-disc configuration.

Tribological tests were configured for reciprocating motion using an Anton Paar TRB^3^ tribometer. The choice of the motion type was based on a literature analysis, which showed that human joints work in a characteristic motion [27] that can be modelled as reciprocating motion. Figure 2 shows the friction pair diagram.

Table 2 compiles the test parameters. The counter-sample in the tested friction pairs was a ball 6 mm in diameter made of Al_2_O_3_ (III) with an Ra equal to 0.32 µm. The tests were repeated five times for each friction pair with the given parameters. The chemical composition of the lubricant used is summarized in Table 3.

Bovine serum is the recommended lubricant for wear testing, but its poor availability and rapid oxidation influenced our decision to use Ringer’s solution.

A combined confocal and interferometric profiler Leica DCM8 was used to measure the post-implantation geometric structure of the surface. The axonometric images, surface profile, and essential amplitude parameters are given in Section 3.1. 

Observations of the surface morphology, cross-sections of the samples, and linear analyses of the implanted layer chemical composition were performed using a Phenom XL scanning electron microscope equipped with an EDS energy dispersion spectrometer. The accelerating voltage was 15 kV and the magnifications used were ×1000, ×3000 and ×5000. Test surface micrographs before tribological tests are shown in Section 3.2, whereas Section 3.3 illustrate linear analyses of the implanted layers.

The hardness of the tested materials was determined by instrumented indentation using an Anton Paar ultra-nanoindentation tester with a Berkovich indenter tip geometry and a radius of ~100 nm. The velocity of the loading force increase was 2 mN/min (the force increased linearly as a function of time). A load of 1 mN was applied. Once the maximum force setpoint was reached during the test, the force was reduced at the same rate as the increasing force until the indenter extended entirely above the sample surface. For the load–unload cycle, a graph of indenter load versus penetration depth was generated. The hardness test results are shown in Section 3.4. The mechanical properties were assessed using the Olivier Pharr method, according to which [28,29]:

  (1) $$E=\frac{E_{i}\left(1-v^{2}\right)}{\frac{2\;*\sqrt{A}}{\sqrt{\pi }\;*\;S}*E_{i}-\left(1-v_{i}^{2}\right)}$$
  where:
*E*, *v*—Young’s modulus and Poisson’s ratio of the tested material;
*E_i_*, *v_i_*—Young’s modulus and Poisson’s ratio of the indenter’s material;
*S*—contact stiffness (tangent of the inclination angle of the unloading curve);
*A*—contact area calculated from the contact depth and indenter’s geometry calibration.

The results of tribological tests are shown in Section 3.5. The chart shows friction coefficient μ as a function of the number of recorded friction pair cycles. The Leica DCM8 confocal microscope in interferometric mode was used to examine the geometric structure of wear tracks after tribological tests on the samples and counter-samples (Section 3.6). Axonometric images, profiles, and wear depths on the cross-section were obtained from the tests. Optical measurements also allowed the determination of the wear mechanism of the friction pairs. Observations of the surface morphology and wear tracks after friction were performed using a Phenom XL scanning electron microscope. The results are shown in Section 3.7.

Contact angle measurements were performed using the Attension Theta tensiometer. The static contact angle was determined in a procedure involving the precise placement of droplets of distilled water (approx. 5 µL) on the sample surface, followed by immediate measurement. Analysis was performed automatically by the software. The droplets were applied to the disc in different parts of the sample. The measurement was repeated five times. The contact angle is an angle formed by intersecting tangent planes at the liquid–solid interface. A surface is hydrophilic (high wettability) when its static contact angle is <90° and hydrophobic (low wettability) when the contact angle is >90°. Biomaterials used as cardiovascular system implants (e.g., stents) should be both hydrophobic and non-thrombogenic for the continuous movement of fluid connective tissue–blood. The contact angle measurements are shown in Section 3.8.

## 3. Results

### 3.1. Confocal Microscopy Results

**Figure 3:**
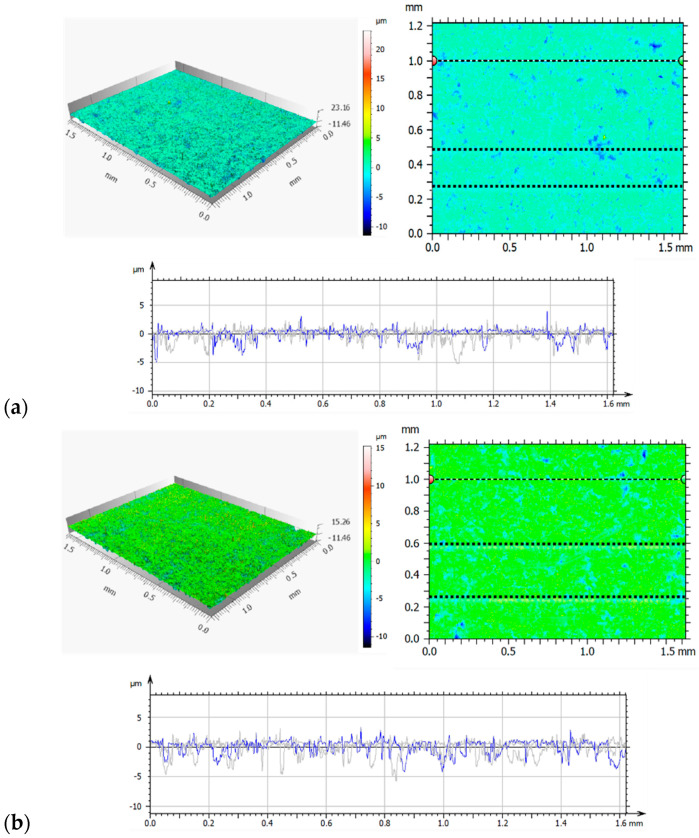

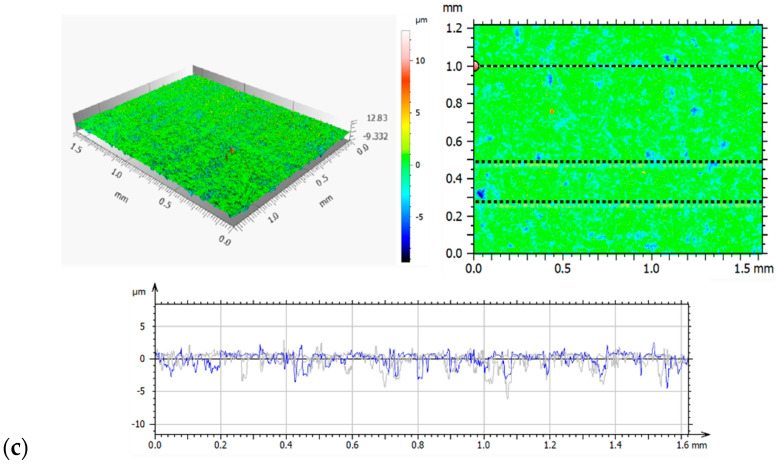
Axonometric images and surface profiles of the reference sample (**a**) and the samples implanted with nitrogen ions (**b**) and oxygen ions (**c**).

A detailed analysis of the surface geometric structure is based on an informed selection of amplitude parameters, which are a valuable source of information on the design and operation of the surfaces tested [19,30]. However, the assessment of surface topography based on only one parameter—*Sa* (arithmetic mean height)—provides insufficient information on the measured profile. Thus, the parameters *Sp*—maximum peak height; *Sv*—maximum valley depth; *Sz*—maximum height of the surface; *Sq*—squared mean height; *Ssk*—asymmetry coefficient (skewness); and *Sku*—flattening (clustering) coefficient (kurtosis) were used in the analysis. These parameters are more sensitive to the presence of valleys and peaks. Figure 3 and Table 4 show the axonometric images, surface profiles, and amplitude parameters of the reference and the implanted samples.

The analysis of the geometrical structures of the reference surface and the implanted samples revealed that the values of all parameters (*Sp*, *Sv*, *Sz*, *Sa*, and *Sq*) were lower than those of the reference sample. These lower values indicate that implantation ensures smooth surfaces. A positive value of *Ssk* informs us of the presence of steep ridges and peaks with sharp tips on the surface of the reference sample. A decrease in the value of *Ssk* to −0.67 in the case of a sample implanted with nitrogen ions indicates a gradual loss of sharpness—an increase in the curvature radius of the tips. The analysis of the amplitude parameters of the sample implanted with oxygen ions showed that its surface was a plateau with gentle slopes and rounded tips. These surface features have a direct influence on the wear mechanism and wear intensity of the tested components.

### 3.2. Scanning Electron Microscopy Results from Surface Morphology Observations

**Figure 4:**
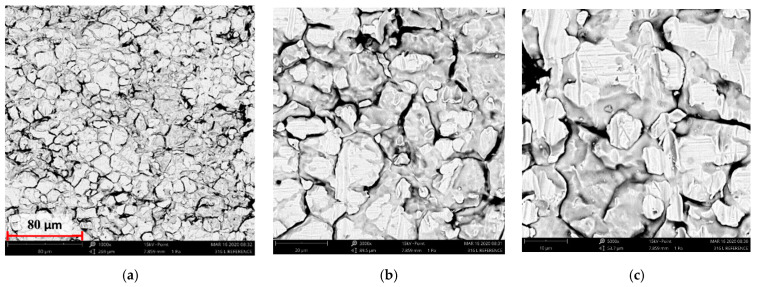
SEM image of the reference sample—316L: (**a**) ×1000, (**b**) ×3000 (**c**) ×5000.

**Figure 5:**
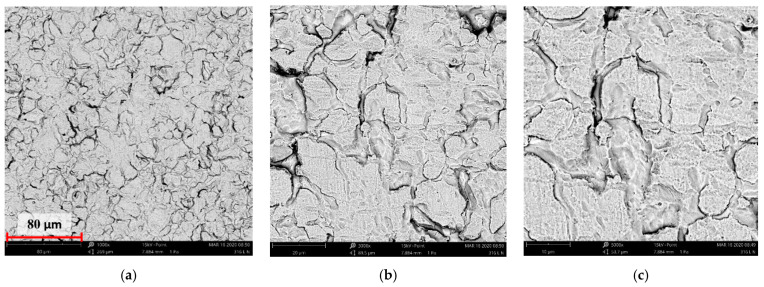
SEM image of the sample implanted with nitrogen ions: (**a**) ×1000, (**b**) ×3000, (**c**) ×5000.

**Figure 6:**
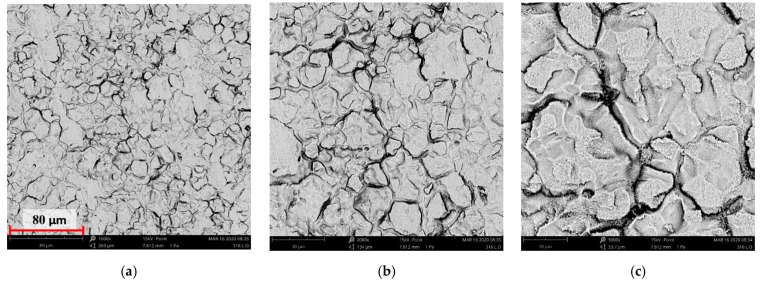
SEM image of the sample implanted with oxygen ions: (**a**) ×1000, (**b**) ×3000, (**c**) ×5000.

**Figure 7:**
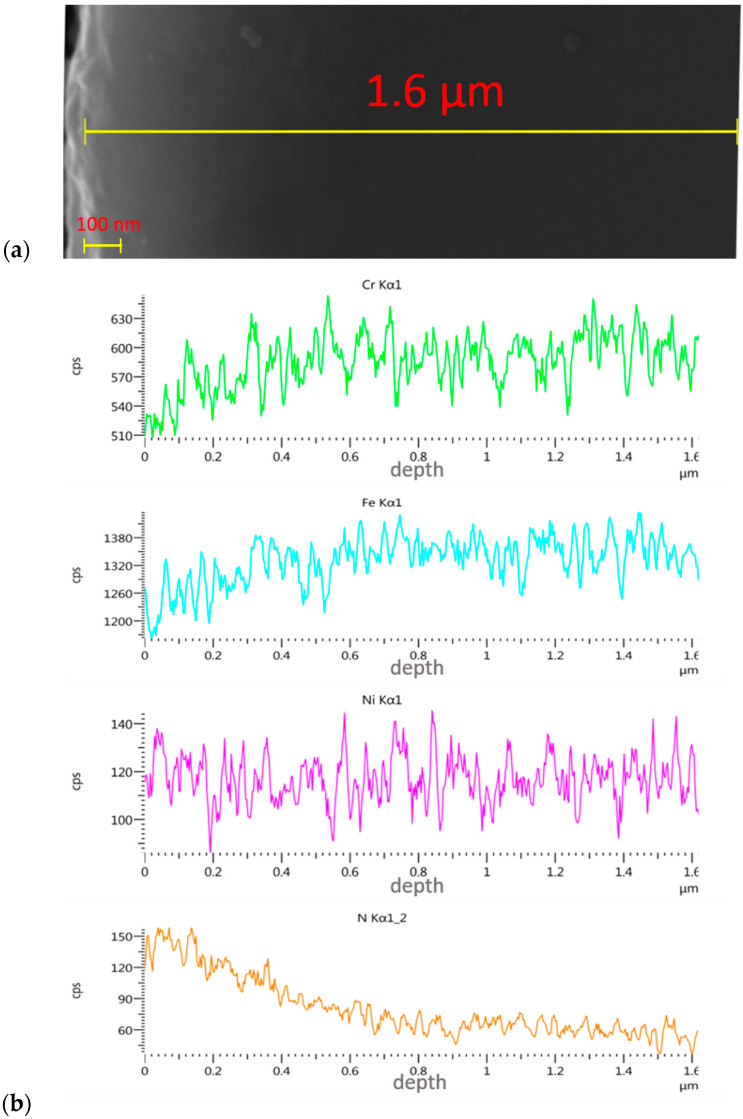
SEM micrograph of the cross-section of steel 316L sample implanted with nitrogen ions (**a**) with linear EDS analysis of elemental distribution (**b**).

**Figure 8:**
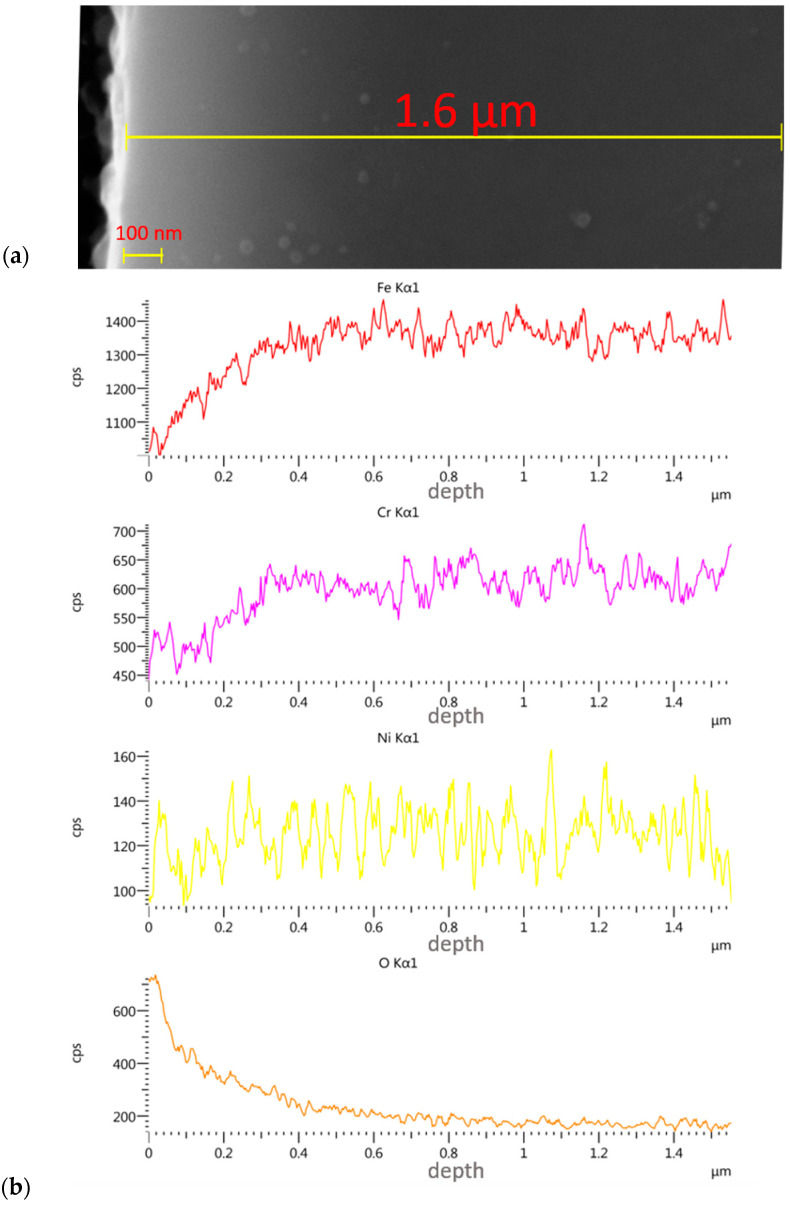
SEM micrograph of the cross-section of steel 316L sample implanted with oxygen ions (**a**) with linear EDS analysis of elemental distribution (**b**).

Figure 4, Figure 5, Figure 6, Figure 7 and Figure 8 show the SEM microstructure images (Figure 4, Figure 5 and Figure 6) and the results of the quantitative analyses of the chemical compositions (Figure 7 and Figure 8).

The observations of the microstructures shown in Figure 4, Figure 5 and Figure 6 reveal the granular characteristics of all samples. Granularity was more pronounced in the reference sample than in those implanted with nitrogen and oxygen ions. In addition, a higher etched area void fraction was observed on the surface implanted with nitrogen ions. As a result, the surface became more homogeneous, as confirmed by the geometric structure examination.

### 3.3. Assessment of Implanted Layers

Thickness assessment involved the preparation of a metallographic section on the transverse cross-section. The thickness of the implanted layer was difficult to evaluate, as the boundary between the implanted layer and the substrate was not clear. Only brighter and darker zones were observed, and these were impossible to measure. The depth of the nitrogen and oxygen ion implantation was determined through linear analyses.

The gradual change in colour from brighter (upper part of the layer) to the darker (lower part of the layer) indicates the typical nature of the layers modified by ionic implantation—i.e., the lack of a clear boundary between the modified surface layer and the 316L core. From Figure 7 and Figure 8, it follows that the nitrogen ions penetrated the sample to a depth of 500 nm at the same process parameters, with an average effective penetration range of 180 nm. In the case of the oxygen ions, the maximum penetration depth was about 350 nm, with an average range of about 60 nm. The percentage content of other elements constituting the alloy increased with depth and the loss of the implanted layer.

### 3.4. Nanohardness of Deposited Layers

**Figure 9:**
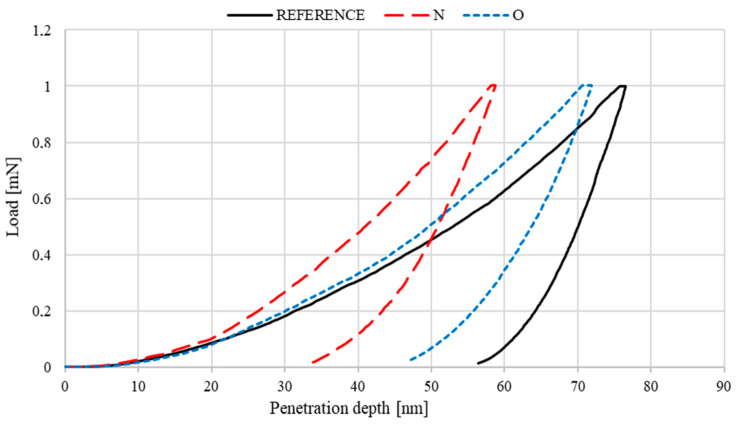
Loading–penetration depth curve from nanohardness tests.

Figure 9 shows an example penetration depth curve for a nominal loading force of 1 mN with a marked maximum force for which h_max_ indentation is determined. The penetration depth measurements were the basis for determining the most important mechanical parameters. Table 5 compiles the mean values of the parameters obtained from five measurements.

The curves presented in Figure 9 indicate that, compared to the reference sample, the deposited layers are more elastic, as shown by the indentation curve slope and the plastic and elastic behaviour values. Moreover, the lower Wtot value of the implanted samples proves that the deposited layers are less susceptible to deformation due to service loads. The instrumented hardness tests clearly showed improved hardness after implantation. Nitrogen ion implantation provided an approximately 45% higher efficiency and a 20% increase in the Young’s modulus values. Oxygen ion implantation increased the Young’s modulus values by about 5%. The same increase can be observed in the contact area values. The nanohardness results indicate that the nitrogen ion-implanted samples should have the most beneficial tribological characteristics. 

### 3.5. Tribological Test

**Figure 10:**
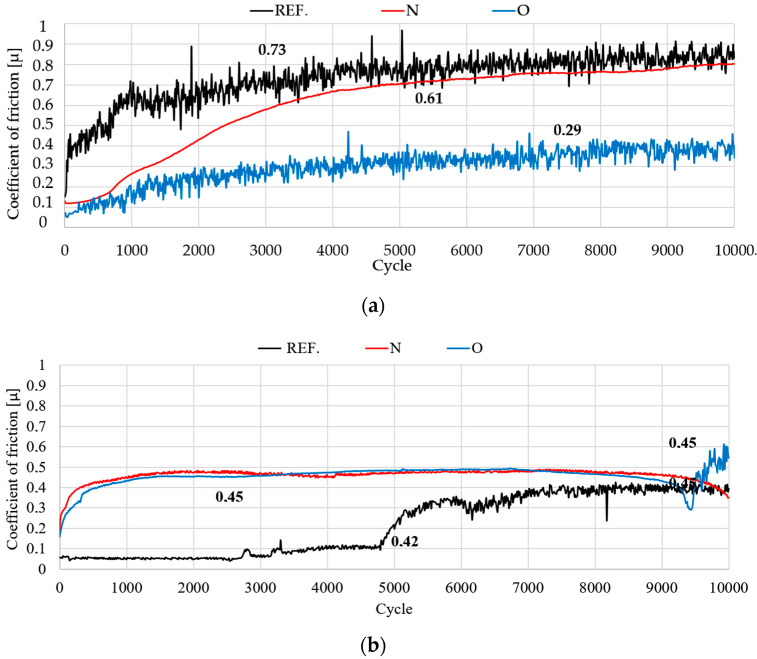
Average friction coefficient obtained (**a**) under dry friction (DF) and (**b**) with the use of Ringer’s solution (RS) as a lubricant.

Figure 10a,b show the plots of friction coefficient μ as a function of the number of recorded friction pair cycles. The values on the graph are averaged values of the friction coefficient measured during three measurement series.

The results of the tribological tests indicate that, under dry friction, the implanted samples displayed the lowest resistance to motion. The average coefficients of friction were comparable in all samples when Ringer’s solution was used as a lubricant. During dry sliding, a rapid increase in the friction coefficient was observed for the nitrogen ion-implanted sample between cycles 1 and 5000, after which the increase became less rapid. In the final stage, the maximum coefficient value was about 0.61. Compared to the reference sample, the coefficient’s mean value decreased by about 20%. Under lubricated conditions, the reference sample recorded the lowest resistance to motion. During cycle 5000, the friction coefficient rose sharply from 0.06 to about 0.4, most likely due to the wear debris present at the sample – ball interface. The wear debris was present until the end of the test, as indicated by the unchanged value of the µ parameter, which was 0.42.

### 3.6. Assessment of Surface Geometric Structure of Samples and Counter-Samples

**Figure 11:**
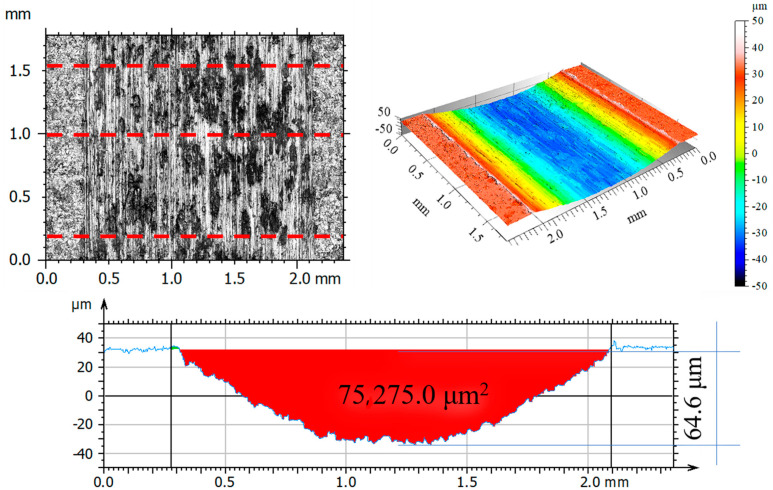
Optical and axonometric views of sample wear track and the wear profile on transverse cross-section after the dry sliding of the steel 316L—Al_2_O_3_ friction pair.

**Figure 12:**
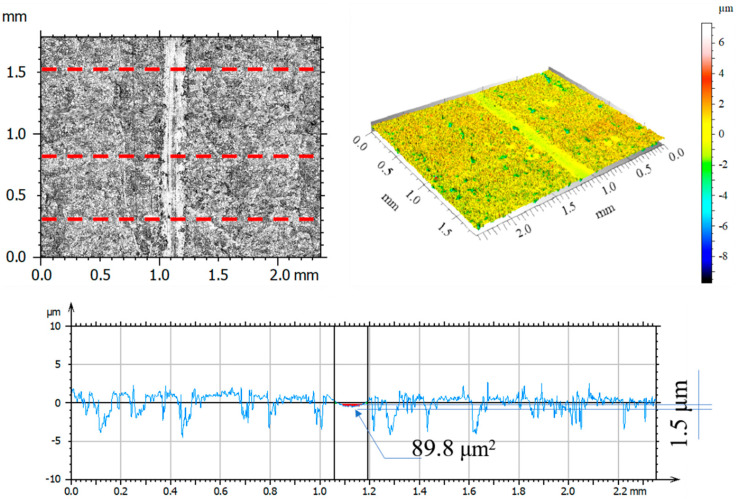
Optical and axonometric views of sample wear track and the wear profile on transverse cross-section after the dry sliding of the nitrogen ion-implanted steel 316L—Al_2_O_3_ friction pair.

**Figure 13:**
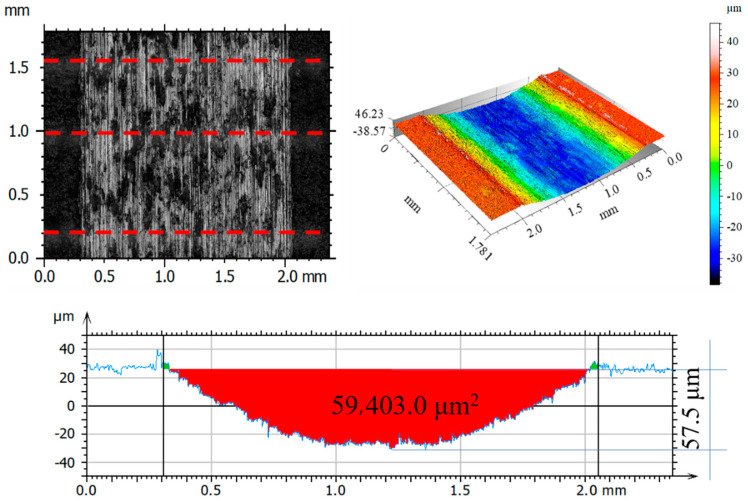
Optical and axonometric views of sample wear track and the wear profile on transverse cross-section after the dry sliding of the oxygen ion-implanted steel 316L—Al_2_O_3_ friction pair.

**Figure 14:**
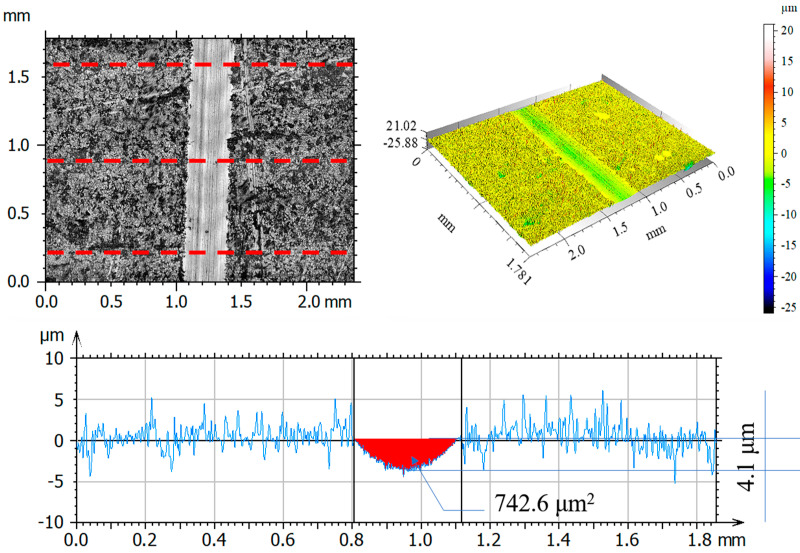
Optical and axonometric views of sample wear track and the wear profile on transverse cross-section after the RS-lubricated sliding of the steel 316L—Al_2_O_3_ friction pair.

**Figure 15:**
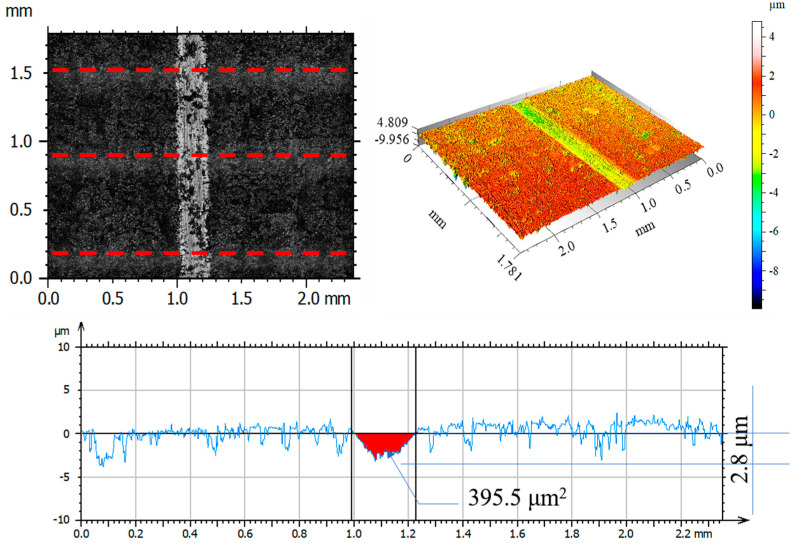
Optical and axonometric views of sample wear track and the wear profile on transverse cross-section after the RS-lubricated sliding of the nitrogen ion-implanted steel 316L—Al_2_O_3_ friction pair.

**Figure 16:**
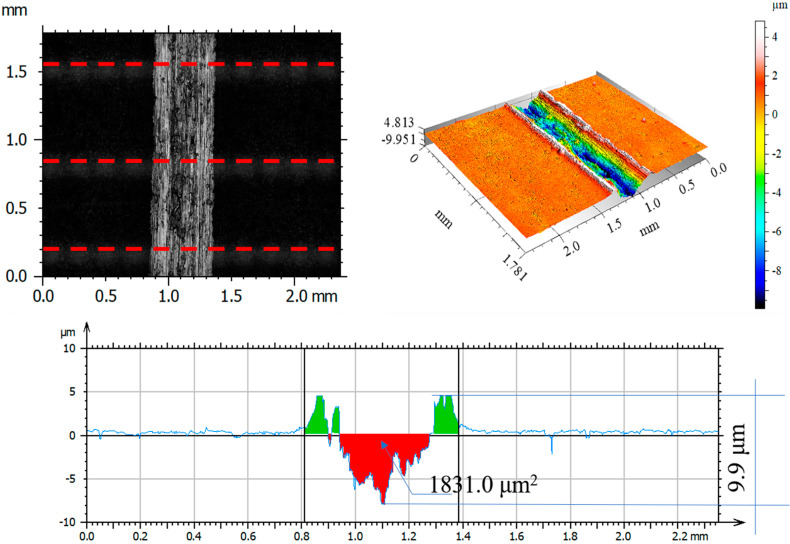
Optical and axonometric views of sample wear track and the wear profile on transverse cross-section after the RS-lubricated sliding of the oxygen ion-implanted steel 316L—Al_2_O_3_ friction pair.

**Figure 17:**
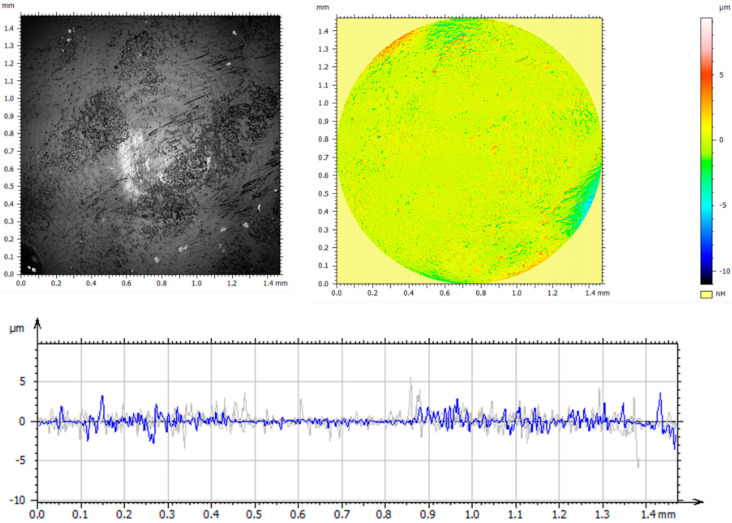
Optical and axonometric views of ball wear track and the wear profile on transverse cross-section after the RS-lubricated sliding of the steel 316L—Al_2_O_3_ friction pair.

**Figure 18:**
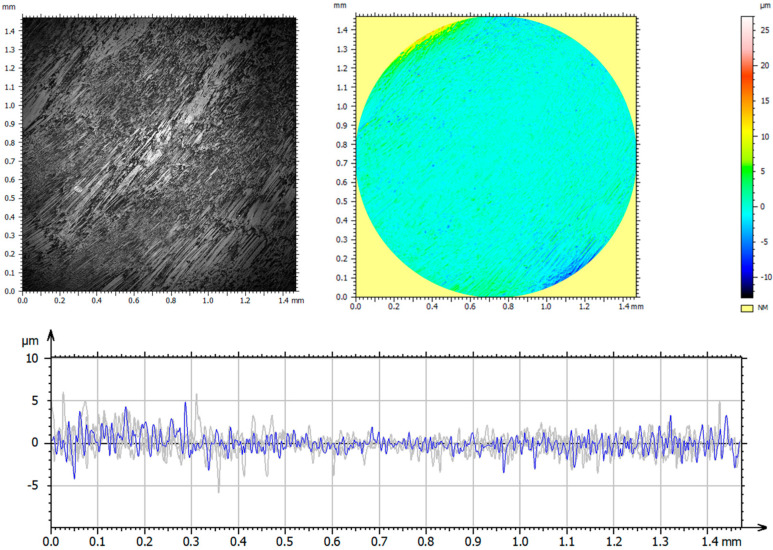
Optical and axonometric views of ball wear track and the wear profile on transverse cross-section after the RS-lubricated sliding of the nitrogen ion-implanted steel 316L—Al_2_O_3_ friction pair.

**Figure 19:**
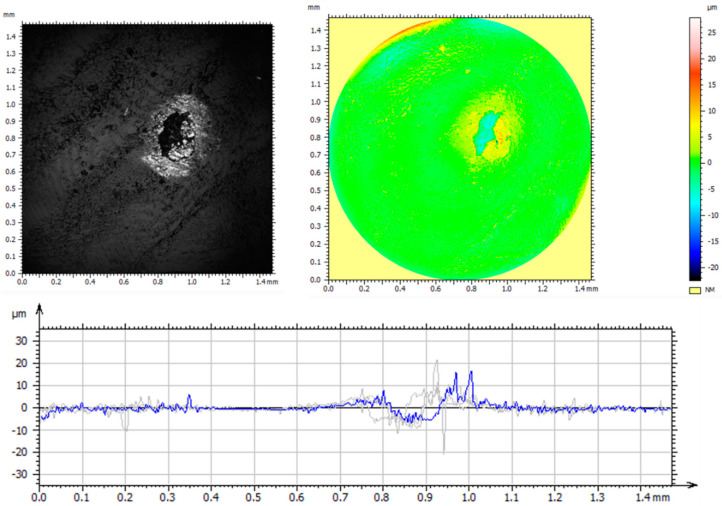
Optical and axonometric views of ball wear track and the wear profile on transverse cross-section after the RS-lubricated sliding of the oxygen ion-implanted steel 316L—Al_2_O_3_ friction pair.

After tribological tests, the wear tracks on the samples and counter-samples were measured and average depths and wear areas of the samples and counter-samples (balls) were determined from three series of measurements. The test results are shown in Figure 11, Figure 12, Figure 13, Figure 14, Figure 15, Figure 16, Figure 17, Figure 18 and Figure 19. Table 6 and Table 7 summarise the amplitude parameters of the wear tracks.

After tribological tests, the analysis of the surface geometric structure revealed the fastest wear rates in the reference sample and in the sample implanted with oxygen ions under both dry and lubricated friction. Despite the lower resistances to motion obtained when Ringer’s solution was used, the wear of the samples was several times higher compared to that of the nitrogen ion-implanted material. Microscopic examination revealed an abrasive wear mechanism in all cases under analysis.

Figure 17, Figure 18 and Figure 19 show examples of optical and axonometric wear track images for a counter–Al_2_O_3_ ball pair. For sliding with RS lubrication, the highest counter-sample wear was recorded in friction pairs 316L—Al_2_O_3_ and 316L O^+^—Al_2_O_3_, where the ball wear was 138.9 μm^2^ and 427.3 μm^2^, respectively. For 316L N^+^, the ball wear was 65% lower than that of the reference sample and over 80% lower compared to that of the oxygen ion-implanted sample. The analysis of the amplitude parameters showed an increased roughness of the friction surfaces (samples and balls) due to the tests.

### 3.7. Assessment of Wear Mechanism

**Figure 20:**
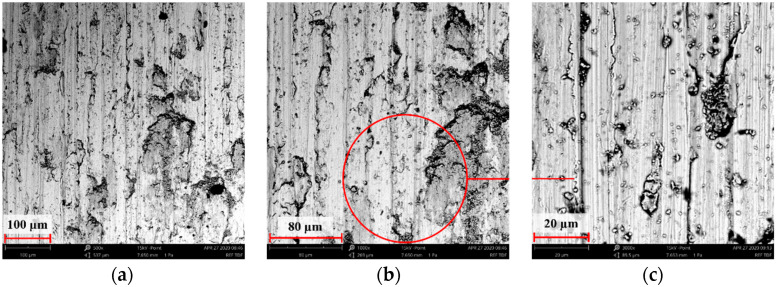
SEM image of the steel 316L wear track after dry sliding: (**a**) ×500, (**b**) ×1000, (**c**) ×3000.

**Figure 21:**
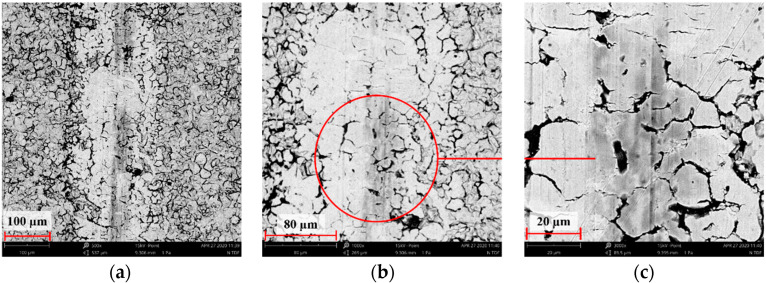
SEM image of the nitrogen ion-implanted steel 316L wear track after dry sliding: (**a**) ×500, (**b**) ×1000, (**c**) ×3000.

**Figure 22:**
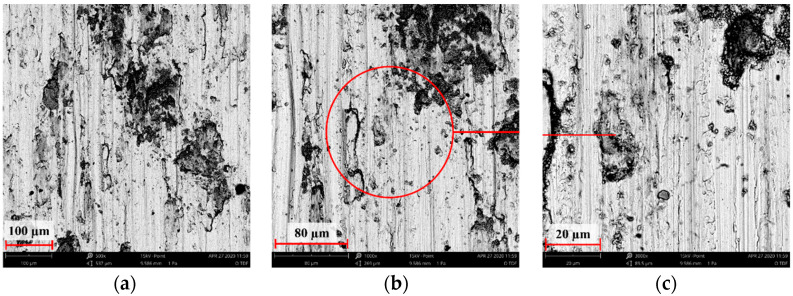
SEM image of the oxygen ion-implanted steel 316L wear track after dry sliding: (**a**) ×500, (**b**) ×1000, (**c**) ×3000.

**Figure 23:**
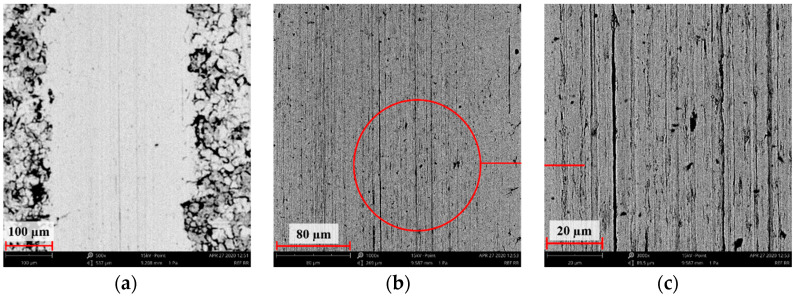
SEM image of the steel 316L wear track after RS-lubricated friction: (**a**) ×500, (**b**) ×1000, (**c**) ×3000.

**Figure 24:**
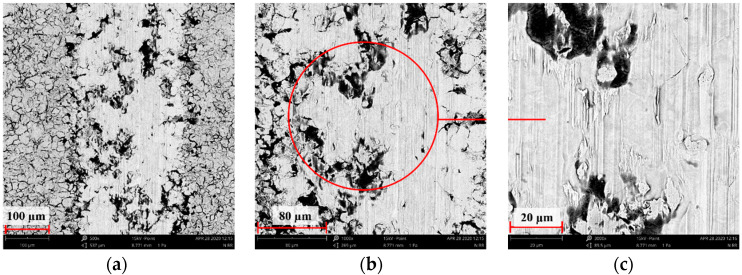
SEM image of the nitrogen ion-implanted steel 316L wear track after friction with RS lubrication: (**a**) ×500, (**b**) ×1000, (**c**) ×3000.

**Figure 25:**
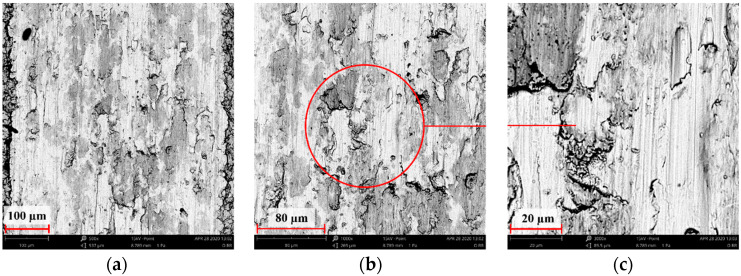
SEM image of the oxygen ion-implanted steel 316L wear track after friction with RS lubrication: (**a**) ×500, (**b**) ×1000, (**c**) ×3000.

To conduct a more detailed analysis and identify the wear mechanisms inovlved, SEM observations of wear tracks were performed. The results are compiled in Figure 20, Figure 21, Figure 22, Figure 23, Figure 24 and Figure 25.

Wear track surface analysis indicated abrasive wear as a dominant mechanism in the reference sample. Ploughing and cutting resulting from secondary wear debris displaced between the interacting surfaces increased wear intensity. The moving wear particles caused cracks or plastic deformations in the form of grooves. More or less intense traces of wear occurred on all tested samples, with the most pronounced tracks (wide and deep) observed on the reference surfaces. 

Far narrower and shallower tracks were observed on the implanted samples, as confirmed by examining the surface geometric structure. SEM images of the deposited layers indicate that the agglomerates of wear debris were pressed into the sample surface during the frictional wear tests. A comprehensive analysis of the surface geometric structure after tribological tests demonstrated that the implanted layer was not removed in the case of the sample ion-implanted with nitrogen.

### 3.8. Contact Angle

**Figure 26:**
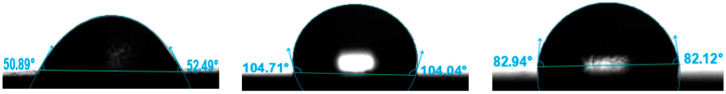
An example of contact angle.

Figure 26 presents an example of the contact angles of the tested surfaces using distilled water. Mean values of the recorded contact angles for the applied measuring liquid are shown in Table 8.

Contact angle measurements confirmed the effect of ion implantation on wettability. The tests showed that 316L and 316L O^+^ steels are hydrophilic. The highest contact angles were displayed by the sample implanted with nitrogen ions. They were about 50% larger compared to the reference sample and about 40% larger compared to the sample implanted with oxygen ions. In the case of cardiovascular implants, the hydrophobicity of the surface is desirable for the continuous movement of fluid connective tissue–blood.

## 4. Conclusions

The following conclusions were formulated based on the test results:

1. The use of ion implantation had a positive effect on the geometric structure of the implanted surface, which became smooth as a result of the process. The smoothing was demonstrated both by the values of all amplitude parameters and the results of the microstructure tests.
2. The linear EDS analysis of the elemental distribution demonstrated that, with the same implantation parameters (5 × 10^17^ N^+^/cm^2^ and 5 × 10^17^ O^+^/cm^2^ at an energy of 35 kV), the nitrogen ions were implanted to a depth of about 500 nm and the oxygen ions to a depth of about 350 nm.
3. Mechanical tests showed a beneficial effect of the proposed process on both hardness and Young’s modulus. Implantation with nitrogen ions provided the highest effectiveness—an approximately 45% increase in hardness and 20% modulus reduction. In addition, the load–penetration depth curves indicated the greater elasticity of the implanted layers compared to those of the reference sample.
4. Under dry friction conditions, implanted samples displayed the most beneficial tribological characteristics. The friction coefficient mean values were about 17% lower when nitrogen ions were used and about 60% lower for oxygen ions. The coefficients of friction were comparable in the case of RS lubricated friction.
5. The analysis of the surface geometric structure after tribological tests showed that the reference sample was the most worn material in the friction pair with Al_2_O_3_ (III). Moreover, the wear of the sample implanted with oxygen ions was also high despite displaying the lowest resistance to motion.
6. The microstructure analysis of wear tracks identified abrasive wear as the dominant wear mechanism in the case of the reference sample and that implanted with oxygen ions. The wear tracks were much wider and deeper compared to the 316L N^+^ sample. The dislodging of loose wear products between friction surfaces resulted in cracks and grooves.

## Figures and Tables

**Table 1:** Chemical composition of type 316L steel.

Alloy 316L [% Content]									
**Fe**	**Cr**	**Ni**	**Mo**	**C**	**Si**	**Mn**	**P**	**S**	**N**
Ba-lance	16.5–18.5	10.0–13.0	2.0–2.5	<0.03	<1.0	<2.0	<0.045	<0.015	<0.011

**Table 2:** Test parameters.

Parameter	Unit	Friction Pair
		Al_2_O_3_ Ball—316L (Reference) Al_2_O_3_ Ball—316L Implanted with N^+^ Ions Al_2_O_3_ Ball—316L Implanted with O^+^ Ions
Load	N	5
Linear viscosity	m/s	0.0159
Cycles	-	10,000
Frequency	Hz	1
Humidity	%	50 ± 1
Temperature	°C	23 ± 1
Lubrication	-	no lubrication (DF) Ringer’s solution (RS)

**Table 3:** Chemical composition of Ringer’s solution.

Chemical Composition [g/dm^3^]		
NaCl	KCl	CaCl_2_
8.6	0.3	0.243

**Table 4:** Amplitude parameters.

Parametr	Unit	Sample		
		316L	316L N^+^	316L O^+^
Sp	μm	23.16	12.83	15.26
Sv	μm	12.46	9.33	10.46
Sz	μm	35.68	22.16	25.72
Sa	μm	0.70	0.75	0.82
Sq	μm	1.7	1.03	1.14
Ssk		1.48	-0.67	0.96
Sku		9.20	7.67	8.06

**Table 5:** Mechanical parameters.

Parameter	Unit	Sample					
		316L		316L N^+^		316L O^+^	
		Mean	SD	Mean	SD	Mean	SD
Instrumented hardness [H_IT_]	GPa	6.2	0.1	11.2	0.2	7.0	0.2
Young’s modulus [E_IT_]	GPa	195.4	21.9	231.2	39.9	200.0	9.3
Contact area [A_p_]	μm^2^	0.162	0.003	0.089	0.022	0.143	0.056
Plastic behaviour [W_plast_]	pJ	17.8	2.3	10.6	3.7	14.4	1.6
Elastic behaviour [W_elast_]	pJ	8.1	1.0	9.2	0.7	9.0	0.6
Total behaviour [W_tot_]	pJ	25.9	1.3	19.8	4.4	23.4	2.3
Maximum indentation [h_m_]	nm	76.6	0.1	58.8	5.9	72.6	0.1
Depth of indenter-sample contact at F_max_ [h_c_]	nm	68.4	0.8	48.6	5.5	63.6	1.5

**Table 6:** Amplitude parameters of wear tracks.

Parameter	Unit	Sample					
		316L		316L N^+^		316L O^+^	
		DF	RS	DF	RS	DF	RS
Sp	μm	58.04	13.24	6.97	3.66	59.0	10.55
Sv	μm	33.64	18.57	7.45	4.51	36.21	11.16
Sz	μm	91.69	1.81	14.43	8.17	95.21	21.71
Sa	μm	18.58	0.99	0.49	0.72	15.90	3.57
Sq	μm	21.34	1.23	0.74	0.85	18.23	4.22
Ssk		0.62	1.10	-2.16	0.6	0.652	0.05
Sku		2.07	4.73	16.94	2.53	2.14	2.12

**Table 7:** Average parameters of the ball surface geometric structure after tribological tests with lubrication.

Parameter	Unit	Friction Pair			
		Al_2_O_3_ before Test	316L— Al_2_O_3_ after Test	316L N^+^— Al_2_O_3_ after Test	316L O^+^— Al_2_O_3_ after Test
Rp	μm	1.67	2.56	2.26	2.81
Rv	μm	1.94	2.87	1.88	3.86
Rz	μm	3.58	5.43	4.14	6.50
Ra	μm	0.32	0.65	0.39	0.68
Rq	μm	0.49	0.87	0.54	0.99
Rsk		−0.54	−0.26	0.26	−0.71
Rku		9.78	4.42	7.48	5.85

**Table 8:** Mean contact angle with distilled water.

	Contact Angle [°]	
	Mean	SD
316L	51.69	0.58
316L N^+^	103.73	1.25
316L O^+^	82.53	0.85

## Data Availability

Not applicable.
